# Relationship between Spiritual Care Competence, Perceived Professional Benefit, and Retention Intention among Intern Nursing Students: A Correlational Study

**DOI:** 10.1155/2023/1184756

**Published:** 2023-12-31

**Authors:** Jing Liu, Weinan Lu, Dan Li, Ting Fang, Yiying Zhang, Yanjia Li, Xiaoying Zeng, Jue Wu, Yu Feng, Limei Zhang, Yanli Hu

**Affiliations:** ^1^School of Nursing, Chengdu Medical College, Chengdu, Sichuan 610500, China; ^2^The First Affiliated Hospital of Chengdu Medical College, Chengdu, Sichuan 610500, China; ^3^School of Laboratory Medicine, Chengdu Medical College, Chengdu, Sichuan 610500, China; ^4^Sichuan Taikang Hospital, Chengdu, Sichuan 610213, China; ^5^The First People's Hospital of Ziyang, Ziyang, Sichuan 641300, China; ^6^School of Nursing, Guangzhou Medical University, Guangzhou, Guangdong 511495, China

## Abstract

**Aims:**

We aimed to investigate nursing students' spiritual care competence, perceived professional benefit, and retention intention and to analyze the relationship among these variables.

**Background:**

Nurse shortages are a global issue, and intern nursing students' willingness to remain in the nursing profession is important. Spiritual care can reduce patients' depression and improve their quality of life. Implementing spiritual care can help individuals have satisfying experiences. Perceived professional benefit is a positive emotional experience that is important in retention intention. However, the relationship among spiritual care competence, perceived professional benefit, and retention intention remains unclear.

**Methods:**

In this cross-sectional and correlational study, convenience sampling was used to recruit 266 intern nursing students in 10 hospitals throughout China. An online questionnaire was used to assess their sociodemographic characteristics, spiritual care competence, perceived professional benefit, and retention intention. Statistical analyses included the *t*-test, analysis of variance, Pearson's correlation analysis, and bootstrap analysis.

**Results:**

Intern nursing students' mean scores were 103.35 ± 19.00 for spiritual care competence, 72.88 ± 10.40 for perceived professional benefit, and 19.58 ± 3.37 for retention intention. Spiritual care competence was positively correlated with perceived professional benefit (*r* = 0.545; *p* < 0.01) and retention intention (*r* = 0.149; *p* < 0.05). Perceived professional benefit was also positively correlated with retention intention (*r* = 0.320; *p* < 0.01). Bootstrap analysis showed that perceived professional benefit completely mediates the relationship between spiritual care competence and retention intention.

**Conclusions:**

We found that nursing interns had a moderate level of retention intention, and perceived professional benefit was the mediating variable between spiritual care competence and retention intention. *Implications*. Our study results suggest that nursing administrators and educators should improve nursing interns' spiritual care competence and perceived professional benefit to enhance their willingness to remain in the nursing profession and alleviate nurse shortages.

## 1. Background

As the global population ages and the burden of disease increases, the worldwide demand for healthcare continues to grow. The World Health Organization reported that the global demand for nurses is expected to exceed 9 million by 2030 [1]. However, the current global nursing staff gap is estimated to exceed 4.6 million [2], making nursing staff shortages a critical global public health issue [3]. The shortage of nursing staff not only affects the overall quality of services in the healthcare system but also increases patient mortality, infection rates, medication errors, and hospital stays [4, 5]. Nursing students are the reserve force as the future nursing population. Pregraduation clinical placements can facilitate the transition from student to nurse roles and have a considerable impact on nursing student retention [6, 7]. Therefore, the intention of clinical internship nursing students to remain in the nursing profession is of great importance in relieving the shortage of nurses [8].

The willingness of nursing students to remain in the nursing profession after clinical placement needs to be improved. Zadeh et al. [9] surveyed midwifery students and found that those who entered clinical practice had significantly lower intentions to stay in the midwifery profession than those prior to clinical practice. A study by Chuan [10] showed that nursing students' intention to remain in the profession at the end of their internship was at an intermediate level, with the highest scores for the entry “I would leave nursing if I had other job opportunities.” In addition, since the outbreak of the coronavirus disease 2019 (COVID-19) pandemic, intern nursing students have been on the front lines with other healthcare providers, where they have encountered additional stressors [6]. The mental health of nursing interns has therefore been affected, and their willingness to leave the nursing profession has increased [6, 11], which may further exacerbate the nursing brain drain. As a result, there is a great need for nursing administrators and educators to find strategies and develop programs to improve the willingness of nurse interns to stay in the nursing profession and expand the nursing talent pool in China and around the world.

Herzberg's two-factor theory [12] suggests that motivational factors such as job performance, enjoyment gained from work, feelings of accomplishment at work, and expectations for future development are factors that influence an individual's work motivation and play a major role over time. Studies have also shown that satisfactory clinical practice experiences and feelings play an important role in a student deciding to continue in the nursing profession [7, 13, 14]. In recent years, as an integral part of holistic care, spiritual care has been recognized as a key element of healthcare guidelines (such as national palliative care guidelines) [15–17]. Spiritual care is defined as care that identifies and responds to the human spirit when faced with life-changing events (e.g., birth, trauma, ill health, and loss or grief) and may include the need for meaning; the need for self-worth; expressing oneself; faith-related support including the need for rituals, prayers, or sacraments; or simply the need for a sensitive audience [18]. Spiritual care not only effectively improves patients' quality of life but also helps caregivers have satisfactory job experiences. A meta-analysis showed that spiritual care reduced patients' anxiety and depression and improved their quality of life [19]. Moreover, successfully implementing spiritual care with patients can help nurses feel that they do not have an ordinary job but rather a spiritual calling and even a “divine blessing,” which will allow nurses to gain job satisfaction and maintain a positive attitude toward their work [20]. A previous study [21] also showed that nurses' spiritual care competence (SCC) had a positive correlation with perceived professional benefit; when SCC was stronger, nurses had more positive feelings and reported more satisfactory experiences. Nurses are the primary providers of spiritual care [22]. In 1998, the American Association of College of Nursing proposed that SCC should be cultivated among nursing students [23]. A crucial stage in the development of nursing students' competence is the clinical practicum [24], and it is unclear whether SCC contributes to intern nursing students remaining in nursing profession.

Perceived professional benefit is based on positive psychology, which refers to the emotional state when individuals feel satisfied and positive in perceiving rewards and benefits brought about by their occupation and when individuals agree that their occupation can promote their overall growth [25]. Perceived professional benefit is important for nursing staff to experience positive emotions at work and enhance their willingness to continue in their profession [26]. Studies have shown that nurses' perceived professional benefit can enhance psychological capital and resilience, reduce burnout, improve professional identity, and increase job satisfaction [27, 28]. Several studies have found that nurses' perceived professional benefit can directly influence retention intention and strengthen the willingness of continuing in nursing work [29, 30]. Nursing interns are in an important period of professional adjustment and transition, and their professional perceptions have a significant impact on future career choices and professional development [31, 32]. Currently, little research has been conducted that examines the relationship between intern nursing students' perceived professional benefit and their willingness to stay in the nursing profession.

Retention intention is defined as an individual's conscious willingness to stay in a profession [33]. Nurses' retention intention is a significant predictor of nurse retention and is key to stabilizing nursing teams [34]. In this study, retention intention was defined as the willingness of nurse interns to stay in a nursing position and work in nursing after their internship. As a special group, intern nursing students are undergoing a change in roles from students to nurses, and the willingness of intern nursing students to stay is very important as to whether they choose nursing positions in the future. However, the current situation, influencing factors, and occurrence mechanisms of intern nursing students' retention are still unclear.

Thus far, most related studies have focused on the nurse population and found that retention intentions are associated with a variety of significant factors [35], such as perceived professional benefit [29, 30]. Many studies have explored the factors that influence nurses' perceived professional benefit [36]. However, few studies have considered the impact or role of SCC. On the one hand, when patients' spiritual suffering worsens as a result of illness and the demand for spiritual care increases [37], the extent of spiritual care provided to patients has a direct impact on clinical care quality, patient satisfaction, and job satisfaction [38, 39]. On the other hand, work competence as a positive personality trait is one of the bases of positive psychology [40], and SCC can be regarded as a source of pleasant emotions and experiences. Therefore, based on Herzberg's two-factor theory [12], we hypothesized that SCC can positively influence retention intention. Based on the theory of positive psychology [40] and previous studies [29, 30], we also hypothesized that SCC can positively influence perceived professional benefit and that perceived professional benefit mediates the relationship between SCC and retention intention.

Nursing interns represent an essential reserve for nursing professionals [41]. Surprisingly, few empirical studies have investigated the relationship between SCC, perceived professional benefit, and retention intention among nursing interns, especially in China, where there is a serious shortage of total nursing human resources [42]. Consequently, in this study, we aimed to explore the relationship between SCC, perceived professional benefit, and retention intention among nursing interns. Our findings can help nursing administrators and educators implement effective interventions to improve intern nursing students' retention, increase future nursing staff, alleviate the shortage of nurses, and enhance the quality of clinical care.

Based on the previous literature, the objectives of this study were (1) to investigate the current situation of SCC, perceived professional benefit, and retention intention among intern nursing students; (2) to analyze the correlation among these three variables; and (3) to explore the mediating role of perceived professional benefit between SCC and retention intention.

## 2. Methods

### 2.1. Study Design and Participants

In this cross-sectional and correlational study, convenience sampling was used to recruit 266 intern nursing students from 10 general hospitals in China in July 2020: three hospitals in Jiangsu Province located in the eastern region, five hospitals in Henan Province located in the central region, and two hospitals in Xinjiang Province located in the western region. The inclusion criteria were (1) full-time nursing majors, (2) who had completed their final clinical internship, and (3) who volunteered to participate in the study. Exclusion criteria were (1) nursing students whose internship was interrupted and (2) those with serious mental disorders or organic diseases.

The sample size in this study was obtained using the rough estimation approach [43], in which the sample size is 10 times the number of independent variables. With 16 independent variables in this study, the sample size was calculated to be 176 instances, assuming that 10% of questionnaires would be invalid.

### 2.2. Measurements

Our self-rated online questionnaire comprised a section on sociodemographic characteristics, the Chinese version of the Spiritual Care Competence Scale (C-SCCS), a brief nurses' perceived professional benefit questionnaire (NPPBQ), and a questionnaire for nurse intention to remain employed. Participants' sociodemographic data included age, sex, education, place of birth, whether they were an only child, frequency of spiritual care training, religious, self-evaluation of work, and self-assessment of health status.

Intern nursing students' SCC was measured using C-SCCS, which was translated by Hu et al. [22] from the Spiritual Care Competence Scale [44]. C-SCCS contains 27 items and three dimensions: spiritual care assessment, implementation, specialization, and quality improvement (twelve items); individual and group support (nine items); and attitude and communication regarding patients' spirituality (six items) [22]. This instrument was scored on a five-point Likert scale; the total score for SCC ranged from 27 to 135, with a higher score indicating greater SCC [22]. Scores of 27–56 were classified as the mild-level group, 57–106 as the moderate-level group, and 107–135 as the high-level group. Cronbach's alpha coefficient of C-SCCS was 0.982 in this study.

Intern nursing students' perceived professional benefit was assessed using NPPBQ, which was developed by Hu et al. [45]. This questionnaire consisted of 17 items in five dimensions: positive career perception (three items), good patient-nurse relationship (four items), family and friend identification (three items), sense of belonging to a team (three items), and self-growth (four items) [45]. This instrument was scored on a five-point Likert scale; the total score for perceived professional benefit ranged from 17 to 85, with a higher score indicating a greater perceived professional benefit [45]. Scores of 17–36 were classified as the mild-level group, 37–66 as the moderate-level group, and 67–85 as the high-level group. In this study, Cronbach's alpha coefficient of NPPBQ was 0.980.

Nursing interns' retention intention was assessed using the questionnaire for nurse intention to remain employed, developed by Hong and Lin [46]. The questionnaire consisted of six items. This instrument was scored on a five-point Likert scale; the total score for retention intention ranged from 6 to 30, with a higher score indicating greater nurses' retention intention [46]. Scores of 6–13 were classified as the mild-level group, 14–23 as the moderate-level group, and 24–30 as the high-level group. In this study, Cronbach's alpha coefficient of the scale was 0.702.

### 2.3. Data Collection

We first contacted hospital administrators to obtain their permission and support. Subsequently, with the help of nursing administrators, a link to our electronic questionnaire (created using the Wenjuanxing electronic data collection platform) was distributed to the WeChat group of the student interns that they managed (in China, nursing administrators typically manage student interns by establishing a special WeChat group). Following this, nursing interns who met the inclusion criteria received the questionnaire electronically for completion. The first page of the electronic questionnaire was the informed consent form. Before proceeding to the completion page, participants were asked to read the informed consent form, which included the purpose of the study, time required to complete the survey (10–20 minutes), and inclusion and exclusion criteria, and then to click on “Acknowledgement of Participation in the Study” before proceeding to the official questionnaire completion page. All participants were assured that their participation in the study was strictly voluntary and anonymous. Finally, the data were stored and managed on the Wenjuanxing platform before being collected and processed by the researcher using a personal account and password that met security requirements.

### 2.4. Ethical Considerations

The present study was approved by the Institutional Review Board of the School of Nursing, Jilin University (access number: 2017092701). Before inclusion, all participants were provided with information about the research and research purposes. The participants were asked to complete the online questionnaires independently and anonymously, so their identifying information was not collected and their privacy was strictly protected. The tools used in this study were authorized by their original authors.

### 2.5. Data Statistics

Data were analyzed using IBM SPSS 26.0 (IBM Corp., Armonk, NY, USA). Quantile-quantile plots were used to examine the normal distribution of intern nursing students' SCC, perceived professional benefit, and retention intention. Descriptive analysis was performed for all data; the results were reported as the percentage, mean, and standard deviation. An independent samples *t*-test and analysis of variance were used to compare demographic differences in intern nursing students' retention intention. Pearson correlation coefficients were used to express the correlation between SCC, perceived professional benefit, and retention intention. Model 4 of Hayes' PROCESS macro in IBM SPSS 26.0 was used to perform bootstrapping to test the mediating role of perceived professional benefit in the relationship between SCC and retention intention. The test level (two-sided) was *α* = 0.05.

## 3. Results

### 3.1. Demographics

Of the 270 intern nursing students recruited to participate in this study, 266 finally completed the survey (for a 98.51% response rate). In this study, the average age of the 266 participants was 20.73 ± 1.09 years. The educational level of all participants was an associate degree. Most participants were female (88.7%). Among the participants, 89.1% were not only children (i.e., had siblings), 75.9% were born in rural areas, 56.4% did not attend spiritual care training, 94.4% had no religious beliefs, 59.8% rated their work self-assessment as good, and 45.5% had better self-health assessment (Table 1).

### 3.2. The Differences of Retention Intention Based on Study Variables

A *t*-test and analysis of variance showed significant differences in intern nursing students' retention intention in terms of sex, frequency of spiritual care training, and self-assessment of health status (all *p* < 0.05) (Table 1).

### 3.3. The Level of SCC, Perceived Professional Benefit, and Retention Intention

The levels of the total mean scores of SCC, perceived professional benefit, and retention intention were divided according to Kelley's [47] finding that divisions between the upper and lower 27% of data are most commonly used in project analysis, as well as according to clinical reality. The total mean score of intern nurses' SCC was 103.35 ± 19.00, which was a moderate level. The mean scores of the assessment, implementation, specialization, and quality improvement of spiritual care; individual and group support; and attitude and communication regarding patients' spirituality were 46.68 ± 8.74, 33.27 ± 7.54, and 23.40 ± 4.59, respectively. Of the dimension item mean scores, attitude and communication regarding patients' spirituality had the highest score (3.90 ± 0.77), followed by assessment, implementation, specialization, and quality improvement of spiritual care (3.89 ± 0.73), and then by individual and group support (3.70 ± 0.84).

The total mean score of perceived professional benefit was 72.88 ± 10.40, which was a moderate to high level. The mean scores of the positive occupational perception; good nurse-patient relationship; recognition from families, relatives, and friends; sense of belonging to a team; and self-growth were 12.49 ± 2.27, 17.41 ± 2.61, 12.75 ± 1.96, 12.87 ± 1.96, and 17.37 ± 2.49, respectively. Of the dimension item mean scores, a good nurse-patient relationship had the highest score (4.35 ± 0.65), followed by self-growth (4.34 ± 0.62), sense of belonging to a team (4.29 ± 0.65), recognition from families, relatives, and friends (4.25 ± 0.65), and positive occupational perception (4.16 ± 0.76).

The total mean score of retention intention was 19.58 ± 3.37, which was a moderate level (Table 2).

### 3.4. Relationship between SCC with Perceived Professional Benefit and Retention Intention

The total mean score of SCC was positively correlated with the total mean score of perceived professional benefit (*r* = 0.545; *p* < 0.01) and with the total mean score of retention intention (*r* = 0.149; *p* < 0.05). The total mean score of perceived professional benefit was significantly positively correlated with the total mean score of retention intention (*r* = 0.320; *p* < 0.01) (Table 3).

### 3.5. Mediating Effect of Perceived Professional Benefit between SCC and Retention Intention

**Figure 1:**
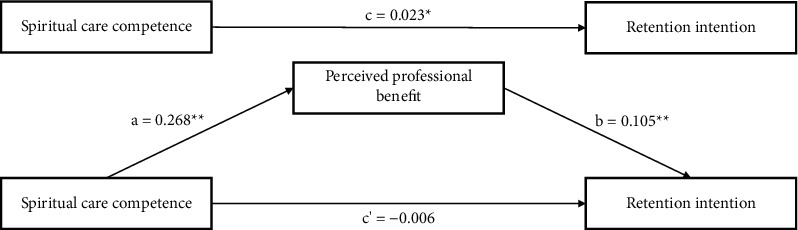
Mediating effect of perceived professional benefit in the relationship between spiritual care competence and retention intention. ^*∗*^*p* < 0.05; ^*∗∗*^*p* < 0.001.

The study assessed the mediating role of perceived professional benefit on the relationship between SCC and retention intention. Control variables were sex, place of birth, whether they were an only child, frequency of spiritual care training, religious, self-evaluation of work, and self-assessment of health status. The results revealed a significant indirect effect of the impact of SCC on retention intention through perceived professional benefit (*b* = 0.028), with a 95% confidence interval (CI) of (0.014, 0.046), which did not contain 0. Furthermore, the direct effect of SCC on retention intention was found to be insignificant (*b* = −0.006), with a 95% CI of (−0.030, 0.019), which did contain 0. Hence, perceived professional benefit completely mediated the relationship between SCC and retention intention. Mediation analysis summary is presented in Table 4 and Figure 1.

## 4. Discussion

This study revealed the level of SCC, perceived professional benefit, and retention intention among nursing interns. We found that perceived professional benefit mediated the relationship between SCC and retention intention. To the best of our knowledge, this was the first study to examine the relationship between these three factors. The results of this study not only provide a better understanding of retention intention among nursing interns but also provide a basis for further research.

First, we examined the status of intern nursing students' SCC, perceived professional benefit, and retention intention based on our findings. In this study, the mean total score for SCC was at a moderate level, which was similar to the findings of a Turkish study [48]. The SCC of intern nursing students in this study was higher than the pre-COVID-19 SCC of Chinese trainee nursing students [49] and Iranian nursing interns [50] and lower than that among Iranian nursing students during the same COVID-19 pandemic period [51]. Possible reasons for this include the following: (1) The threat faced and the particular life-saving process involved during the pandemic facilitated the relationship between nursing staff and patients, patients had greater spiritual care needs, and nursing staff perceived patients' spiritual needs more easily [21]. Past literature [49] used a censored version of the Chinese version of the scale, and different authors have different interpretations and expressions of the Chinese version of the SCC scale. (2) Education and religion have been found to be influencing factors of SCC [52]. Participants in the prior study [51] had undergraduate (bachelor's) degrees, and religious beliefs in Iranian society were fruitful in developing students' spiritual care skills [51]. However, all participants in our study had associate degrees, and only a few (5.6%) reported having religious beliefs. Individual and group support dimensions had the lowest mean scores, probably due to the lack of a uniform spiritual care process in China, lack of multidisciplinary cooperation, work overload, and cultural differences, as well as the fact that many hospitals do not provide spiritual care for patients. In addition, little spirituality training is provided to nursing interns, who have fewer opportunities for clinical education and practice and less knowledge of specific approaches to individual and team support.

This study found that the mean total score of perceived professional benefit among intern nursing students was at a moderate to high level, which was similar to the results of Chinese nursing interns [31, 53]. This may be caused by intern nursing students having accumulated nursing knowledge and experience through clinical practice, enabling them to provide some level of healthcare support for their relatives and friends. Good communication with patients and patients' respect for and reliance on them lead to a good nurse-patient relationship. Cooperation with doctors and other nurses in their work makes nurse interns feel a sense of belonging to a team. Clinical internships translate theoretical knowledge into practical and improve the level of practical operations, promoting interns' growth and thereby enhancing their sense of professional benefit. Because there is little research on perceived professional benefit among nursing interns in countries other than China, we could not make comparisons with other countries. The lowest score was obtained for the dimension of positive career perception. On the one hand, nursing students are prone to negative emotions owing to experiences during the internship, the comments and behaviors of teachers, lack of social respect for nursing staff, and hard work during the night shift [54], leading to poor career perception. On the other hand, during the COVID-19 pandemic, intern nursing students may have experienced fear, dread, and anger in the clinical setting with the risk of exposure to the coronavirus, lack of personal protective equipment, and the possibility of spreading infection to their families [6, 55].

This study found that the mean total score of retention intention among intern nursing students was at a moderate level. Retention intention in the nursing internship was higher in this study than in Chinese nursing interns before the COVID-19 pandemic [10]. This may be because the nursing profession has received greater attention from the state during the pandemic, its social status has improved somewhat, and the professional identity of nursing students has increased [56], leading to their increased willingness to remain in their jobs. Our findings were lower than those in a study from Spain, which reported that a higher percentage of intern nursing students were willing to stay on in the hospital (88.9%) [8]. The reason for this is that China has a significant shortage of total nursing human resources [42]. Because interns and registered nurses care for patients in hospitals together, interns have a greater workload and experience more pressure, which may lead to burnout and reduce their willingness to stay in the profession. Furthermore, in the eyes of the public in China, the social prestige of nurses is much lower than that of doctors [57]. Nursing interns feel this gap and inequality, which affects their willingness to remain in nursing work.

Second, we examined the relationship between nursing interns' SCC, perceived professional benefit, and retention intention based on our findings. We found that nursing interns' SCC is positively correlated with perceived professional benefit, indicating that the higher the ability to provide spiritual care, the greater the perception of professional benefits. This is similar to the findings of a recent study [21]. A qualitative study also found that nurses' professional skills and the value they show in the work process can greatly increase their sense of professional benefit [58]. Spiritual care can reduce patients' anxiety and depression and improve their quality of life [19, 22]. The greater the ability of nursing interns to provide spiritual care, the better they can provide spiritual care to patients in clinical practice, which leads to a sense of self-worth, job accomplishment, and a greater sense of professional benefit. In this study, we found a positive correlation between nursing interns' SCC and retention intention in the workplace, indicating that the higher the SCC, the greater the retention intention. The results were similar to those of past studies [59, 60]. Greater spiritual competencies among nursing interns are associated with a better ability to identify, assess, and meet patients' spiritual needs in the clinical setting; to collaborate with other disciplines and be part of a work team; and to communicate with patients in a spiritual way. This can enrich the nursing student's practice experience, enhance their feelings about practice experiences, and increase satisfaction with practice. Studies have shown that the more satisfied nursing students are with their clinical training, the more willing they are to stay working in the hospital [61]. The results of this study showed a positive correlation between perceived professional benefit and retention intention among nurse interns, indicating that the higher the perceived professional benefit, the higher the retention intention, which is consistent with the literature [29, 30]. This may be because perceived professional benefits can regulate negative emotions, reduce job burnout, help to improve the sense of career identity, and further enhance the willingness to stay in their posts [45].

Finally, a key point is that we found that perceived professional benefit completely mediated the relationship between SCC and retention intention and SCC influenced retention intention through perceived professional benefit. Compared with a previous study that found a positive correlation between care competence and retention intention [59], this study narrowed the scope of care competence to SCC and further found a relationship between SCC and retention intention. Spiritual care is an important component of holistic care and is central to everyday care [15]. As one of the professional nursing skills [62], the stronger SCC, the higher the overall quality of nursing care among intern nursing students and the more likely they are to receive compliments from patients and recognition from their supervisors. This can promote nursing students' satisfaction and positive feelings during clinical practice and enhance their sense of professional benefit. Perceived professional benefit is an intrinsic motivating factor for career development and positive emotional experiences, and the degree of perceived professional benefit can play a key role in nurses' judgments regarding trade-offs and retention, which is an important influencing factor in retention intention [29].

It is suggested that nursing administrators and educators should pay greater attention to the cultivation of intern nursing students' SCC and perceived professional benefit and take measures to enhance SCC in both institutions and hospitals to improve intern nursing students' perceived professional benefit, thereby increasing their retention intentions and reducing brain drain among nurses. Chiang et al. [63] suggested that spiritual education courses should be considered a regular part of the nursing curriculum. For the content of spiritual care education and training programs, Jones et al., in a systematic review, identified key components, including the body of knowledge in spiritual care; self-awareness and use of self; communication and interpersonal relationship in spiritual care; assessment and implementation of spiritual care; and quality assurance in spiritual care [64]. At the same time, education needs to address the weaknesses in SCC. The intern nurses in this study had poor individual and team support, which is similar to the results of an Iranian study [50]; targeted training to strengthen the multidisciplinary team component of spiritual care is recommended so that nursing students can understand how different departments collaborate in referrals. In addition, educators and administrators should explore indigenous Chinese models of spiritual care. For example, by incorporating the cultural characteristics of Confucianism's “benevolence” and Buddhism's “Buddha,” “compassion,” and “goodness,” traditional Chinese thought and culture can be incorporated into spiritual care to form a spiritual care service model that is highly adaptable and suitable for clinical practice to enhance SCC among intern nursing students [49]. Nursing managers and educators should pay greater attention to the professional emotional experience of nursing interns and take measures to enhance their perceived professional benefit, including positive career perceptions, good nurse-patient relationships, family and friend recognition, a sense of belonging to a team, and their own growth [45]. Based on this, it is necessary to strengthen training on career cognition and career planning; actively promote positive nursing experiences to guide intern nurses in developing positive career identity and perception, strengthen training in professional and communication skills, create a collaborative and enthusiastic participatory clinical practice environment and build a collaborative clinical practice model [65] to improve intern nurses' sense of belonging, and provide opportunities for exchange and learning outside the hospital. In addition, our study indicated that the differences in scores for nursing interns' retention intention according to sex and self-assessment of their health status were statistically significant. In our study results, male intern nursing students' retention intention was higher than that of female students, which is inconsistent with the results of previous studies [10, 29]. This may be because during the COVID-19 pandemic, female intern nurses had more negative emotions than male intern nurses [66]. Poor mental health had a significant impact on nurse interns, exacerbating their intention to leave the profession [11]. Nursing managers and educators should attach greater importance to the physical and mental health of nursing interns and provide timely guidance on how to deal with negative emotions and stress [67], especially among female nursing interns.

### 4.1. Limitations

The present study has several limitations. First, while we considered sample representativeness by selecting participants from hospitals in different regions of China, convenience sampling was used in this study, with the data collected in only 10 hospitals in China and the educational level of all participants in our study not including an undergraduate or postgraduate degree, which limits the generalizability of the results. In the future, stratified random sampling should be considered for sample selection. Second, this was a cross-sectional study, and causal relationships between variables could not be determined. In subsequent studies, longitudinal data should be collected to determine the causal relationships between these variables. Finally, the variables in this study were gathered using self-reported methods, which may lead to bias. Future studies should consider adding objective indicators.

## 5. Conclusions

This study showed that retention intention and SCC among Chinese intern nursing students are at a moderate level and perceived professional benefit is at a moderately high level. Our key findings were that SCC and perceived professional benefit have a significant positive correlation with retention intention and that SCC has an impact on retention intention through the mediation of perceived professional benefit. Our results may provide new ideas for hospital and school administrators to develop interventions aimed at improving intern nursing students' retention. In the future, hospital and school administrators can develop more specific and feasible spiritual programs to increase SCC and improve perceived professional benefit, enhancing intern nursing students' willingness to remain in the nursing profession.

### 5.1. Implications for Nursing Management

In light of increased global aging and nursing staff shortages, it is particularly important to understand the retention intention of nursing interns. The results of our study indicate that SCC influences retention intention among nursing interns through the mediation of perceived professional benefit. Our results can provide a new perspective so as to take measures to improve retention intention among nursing interns and reduce nursing brain drain. It is suggested that nursing managers and educators should develop training programs to improve nursing interns' SCC, improve the quality of clinical spiritual care, and contribute to the positive emotions and experiences of nursing interns. It is important to provide education on career awareness and career planning, as well as professional and communication skills training, in addition to developing a collaborative and participatory clinical practice environment and creating more opportunities for learning and communication to enhance perceived professional benefit among nursing interns.

## Figures and Tables

**Table 1:** Comparison of retention intention among intern nursing students with different sociodemographic characteristics (*n* = 266).

Sociodemographic characteristics	Number (%)	Retention intention (mean ± SD)	*t*/*F*	*p*
Sex			2.009	0.046
Male	30 (11.3)	20.73 ± 3.63		
Female	236 (88.7)	19.43 ± 3.32		
Only child			0.427	0.670
Yes	29 (10.9)	19.83 ± 2.98		
No	237 (89.1)	19.54 ± 3.42		
Place of origin			0.803	0.449
Urban	55 (20.7)	19.49 ± 3.84		
Rural	202 (75.9)	19.66 ± 3.28		
Urban-rural	9 (3.4)	18.22 ± 1.92		
Frequency of spiritual care training			2.528	0.041
No	150 (56.4)	19.49 ± 3.26		
Rarely	48 (18.0)	20.13 ± 3.43		
Sometimes	50 (18.8)	18.68 ± 3.05		
Often	14 (5.3)	21.21 ± 3.64		
Always	4 (1.5)	21.75 ± 6.65		
Religious			0.286	0.775
No	251 (94.4)	19.59 ± 3.37		
Yes	15 (5.6)	19.33 ± 3.52		
Work self-evaluation			1.181	0.309
Excellent	59 (22.2)	20.10 ± 3.48		
Good	159 (59.8)	19.33 ± 3.15		
Qualified	48 (18.0)	19.73 ± 3.90		
Self-assessment of health status			3.200	0.014
Very bad	6 (2.3)	16.67 ± 5.65		
Not good	7 (2.6)	17.29 ± 3.86		
Better	121 (45.5)	19.53 ± 3.09		
Good	57 (21.4)	19.23 ± 2.69		
Very good	75 (28.2)	20.36 ± 3.80		

SD, standard deviation.

**Table 2:** Descriptive analyses of spiritual care competence, perceived professional benefit, and retention intention (*n* = 266).

Variables	Min	Max	Mean ± SD (item mean score)
Spiritual care competence			
Assessment, implementation, specialization, and quality improvement of spiritual care	12.00	60.00	46.68 ± 8.74 (3.89 ± 0.73)
Individual and group support	9.00	45.00	33.27 ± 7.54 (3.70 ± 0.84)
Attitude and communication regarding patients' spirituality	6.00	30.00	23.40 ± 4.59 (3.90 ± 0.77)
Total score	27.00	135.00	103.35 ± 19.00 (3.83 ± 0.70)
Perceived professional benefit			
Positive occupational perception	3.00	15.00	12.49 ± 2.27 (4.16 ± 0.76)
Good nurse-patient relationship	4.00	20.00	17.41 ± 2.61 (4.35 ± 0.65)
Recognition from families, relatives, and friends	3.00	15.00	12.75 ± 1.96 (4.25 ± 0.65)
Sense of belonging to a team	3.00	15.00	12.87 ± 1.96 (4.29 ± 0.65)
Self-growth	4.00	20.00	17.37 ± 2.49 (4.34 ± 0.62)
Total score	17.00	85.00	72.88 ± 10.40 (4.29 ± 0.61)
Retention intention			
Total score	6.00	30.00	19.58 ± 3.37 (3.26 ± 0.56)

Min, minimum; Max, maximum; SD, standard deviation.

**Table 3:** Correlation analyses between spiritual care competence, perceived professional benefit, and retention intention (*n* = 266).

Variables	Spiritual care competence	Perceived professional benefit	Retention intention
Spiritual care competence	1		
Perceived professional benefit	0.545^*∗∗*^	1	
Retention intention	0.149^*∗*^	0.320^*∗∗*^	1

^*∗*^ *p* < 0.05; ^*∗∗*^*p* < 0.01.

**Table 4:** Mediating effect of perceived professional benefit between spiritual care competence and retention intention (*n* = 266).

Effects	*B*	Bootstrap SE	Bootstrap 95% CI
Total effect	0.023	0.011	0.001–0.045
Direct effect	−0.006	0.012	−0.030–0.019
Indirect effect	0.028	0.008	0.014–0.046

SE, standard error; CI, confidence interval.

## Data Availability

The data that support the findings of this study are available from the corresponding authors upon reasonable request.

## References

[1] Tsai S. Y. (2020). The nursing profession in a globalizing world. *Journal of Nursing*.

[2] World Health Organization (Who) (2020). *State of the World’s Nursing 2020: Investing in Education, Jobs and Leadership*.

[3] Peters M. (2023). Time to solve persistent, pernicious and widespread nursing workforce shortages. *International Nursing Review*.

[4] Aiken L. H., Simonetti M., Sloane D. M. (2021). Hospital nurse staffing and patient outcomes in Chile: a multilevel cross-sectional study. *Lancet Global Health*.

[5] Nantsupawat A., Poghosyan L., Wichaikhum O. A. (2022). Nurse staffing, missed care, quality of care and adverse events: a cross-sectional study. *Journal of Nursing Management*.

[6] Eweida R. S., Rashwan Z. I., Desoky G. M., Khonji L. M. (2020). Mental strain and changes in psychological health hub among intern-nursing students at pediatric and medical-surgical units amid ambience of COVID-19 pandemic: a comprehensive survey. *Nurse Education in Practice*.

[7] Kaihlanen A. M., Elovainio M., Haavisto E., Salminen L., Sinervo T. (2020). Final clinical practicum, transition experience and turnover intentions among newly graduated nurses: a cross sectional study. *Nurse Education Today*.

[8] Rodríguez-García M. C., Gutiérrez-Puertas L., Granados-Gámez G., Aguilera-Manrique G., Márquez-Hernández V. V. (2021). The connection of the clinical learning environment and supervision of nursing students with student satisfaction and future intention to work in clinical placement hospitals. *Journal of Clinical Nursing*.

[9] Zadeh F. H., Neiterman E., Chowhan J. (2020). Work-life interface and intention to stay in the midwifery profession among pre- and post-clinical placement students in Canada. *Human Resources for Health*.

[10] Chuan J. (2019). *Investigation and Study on the Psychological Capital and Intent to Stay of Nursing Undergraduates in the Post-internship Period*.

[11] Haririan H., Samadi P., Lalezari E., Habibzadeh S., Porter J. E. (2022). Nursing and midwifery students’ mental health status and intention to leave during covid-19 pandemic. *SAGE Open Nursing*.

[12] Alshmemri M., Shahwan-Akl L., Maude P. (2017). Herzberg’s two-factor theory. *Life Science Journal*.

[13] Zhang J., Shields L., Ma B. (2022). The clinical learning environment, supervision and future intention to work as a nurse in nursing students: a cross-sectional and descriptive study. *BMC Medical Education*.

[14] Kim J., Chae D., Yoo J. Y. (2021). Reasons behind generation Z nursing students’ intentions to leave their profession: a cross-sectional study. *Inquiry: The Journal of Health Care Organization, Provision, and Financing*.

[15] Ghorbani M., Mohammadi E., Aghabozorgi R., Ramezani M. (2021). Spiritual care interventions in nursing: an integrative literature review. *Supportive Care in Cancer*.

[16] Balboni M. J., Sullivan A., Amobi A. (2013). Why is spiritual care infrequent at the end of life? Spiritual care perceptions among patients, nurses, and physicians and the role of training. *Journal of Clinical Oncology*.

[17] Lo T. J., Neo P. S. H., Peh T. Y. (2019). Improving quality of palliative care through implementation of national guidelines for palliative care. *Journal of Palliative Medicine*.

[18] van Leeuwen R., Attard J., Ross L. (2021). The development of a consensus-based spiritual care education standard for undergraduate nursing and midwifery students: an educational mixed methods study. *Journal of Advanced Nursing*.

[19] Danna L., Yan Z. (2022). The effect of spiritual care on the quality of life, spiritual health and psychological status in terminal-stage patients:a Meta-analysis. *Journal of Nursing Administration*.

[20] Chen M. L., Chen Y. H., Lin L. C., Chuang L. L. (2020). Factors influencing the self-perceived competencies in spiritual care of nurses in the long-term care facilities. *Journal of Nursing Management*.

[21] Li Y., Zeng X., Chen M. (2022). Association between spiritual care competency and perceived professional benefit among nurses: a cross-sectional study. *Journal of Nursing Management*.

[22] Hu Y., Leeuwen R. V., Li F. (2019). Psychometric properties of the Chinese version of the spiritual care competency scale in nursing practice: a methodological study. *BMJ Open*.

[23] American Association of Colleges of Nursing (1998). *The Essentials of Baccalaureate Education for Professional Nursing Practice*.

[24] Hu S., Chen J., Jiang R. (2022). Caring ability of nursing students pre- and post-internship: a longitudinal study. *BMC Nursing*.

[25] Jing H., Xiaohong L. (2013). Establishment of A Questionnaire of nurses’ perceived professional benefits:reliability and validity assessment. *Military Nursing*.

[26] Wang M., Wang L., Lu C. (2023). Nurses’ sense of organizational support, Self-esteem and perceived professional benefits: a mediating model. *Nursing Open*.

[27] Zhan T., Li H., Ding X. (2020). Can social support enhance sense of coherence and perceived professional benefits among Chinese registered nurses? A mediation model. *Journal of Nursing Management*.

[28] Jinhua Z., Yinghua C., Xiaodong C., Zhenghong X., Longmei D., Qing Z. (2022). Investigation on job satisfaction, humanistic practice ability and perceived professional benefit of nursing staff. *Chinese Evidence-Based Nursing*.

[29] Min F., Yanxian H., Haiyang Y., Tao G., Fei Y. (2021). Construction of the structure equation model of nurses’ psychological capital, occupational benefit and willingness to stay. *Journal of Nurses Training*.

[30] Liu X., Ju X., Liu X. (2021). The relationship between resilience and intent to stay among Chinese nurses to support Wuhan in managing COVID-19: the serial mediation effect of post-traumatic growth and perceived professional benefits. *Nursing Open*.

[31] Hong W., Xia C., Si-qi L., Xing W. (2022). Mediating effect of emotional intelligence on perceived occupational benefit and job adjustment disorder of intern nursing students in Urumqi. *Occupation and Health*.

[32] Liu L., Lv Z., Zhou Y., Liu M., Liu Y. (2023). The mediating effect of the perceived professional benefit of new nurses in cancer hospitals on the nursing work environment, psychological resilience, and transition shock: a cross-sectional questionnaire survey. *Journal of Nursing Management*.

[33] Tett R. P., Meyer J. P. (1993). Job satisfaction, organizational commitment, turnover intention, and turnover: path analyses based on meta‐analytic findings. *Personnel Psychology*.

[34] Brown P., Fraser K., Wong C. A., Muise M., Cummings G. (2013). Factors influencing intentions to stay and retention of nurse managers: a systematic review. *Journal of Nursing Management*.

[35] Pressley C., Garside J. (2023). Safeguarding the retention of nurses: a systematic review on determinants of nurse’s intentions to stay. *Nursing Open*.

[36] Li-yuan S., Xiao-rong L. (2021). Main influencing factors of occupational benefit for Chinese nurses: a Meta-analysis. *Modern Preventive Medicine*.

[37] Harrad R., Cosentino C., Keasley R., Sulla F. (2019). Spiritual care in nursing: an overview of the measures used to assess spiritual care provision and related factors amongst nurses. *Acta BioMedica: Atenei Parmensis*.

[38] Yu J., Gui-zhen F., Shuo M. (2020). Experience of nurses’ spiritual care:a meta-synthesis of qualitative studies. *Chinese Journal of Nursing Education*.

[39] Modderkolk L., van Meurs J., de Klein V., Engels Y., Wichmann A. B. (2023). Effectiveness of meaning-centered coaching on the job of oncology nurses on spiritual care competences: a participatory action research approach. *Cancer Nursing*.

[40] Seligman M. E. (1998). Building human strength: psychology’s forgotten mission. *APA monitor*.

[41] Zhang Z., Zhang X., Fei Y. (2023). Emotional intelligence as a mediator between spiritual care-giving competency and core competencies in Chinese nursing interns: a cross-sectional study. *Supportive Care in Cancer*.

[42] Xiaoqing L. (2021). Study on the cultivation of nursing talents in China based on the profession demands. *Chinese Health Service Managemen*.

[43] ping N., jingli C., na L. (2010). The sample size estimation in quantitative nursing research. *Chinese Journal of Nursing*.

[44] van Leeuwen R., Tiesinga L. J., Middel B., Post D., Jochemsen H. (2009). The validity and reliability of an instrument to assess nursing competencies in spiritual care. *Journal of Clinical Nursing*.

[45] Hu Y., Hu J., Li L., Zhao B., Liu X., Li F. (2020). Development and preliminary validation of a brief nurses’ perceived professional benefit questionnaire (NPPBQ). *BMC Medical Research Methodology*.

[46] Hong T., Lin W. (2010). Establishment of questionnaire for nurse intention to remain employed:the Chinese version. *Academic Journal of Naval Medical University*.

[47] Kelley T. L. (1939). The selection of upper and lower groups for the validation of test items. *Journal of Educational Psychology*.

[48] Sezer T. A., Ozturk Eyimaya A. (2022). Competencies of nursing students in the provision of spiritual care and the factors affecting spiritual caregiving. *Perspectives in Psychiatric Care*.

[49] Mengying Q., Jin Y., Ping M. (2019). The current status of spiritual care ability of nursing interns and improvement countermeasures. *Chinese Nursing Management*.

[50] Babamohamadi H., Tafreshi A., Khoshbakht S., Ghorbani R., Asgari M. R. (2022). Nursing students’ professional competence in providing spiritual care in Iran. *Journal of Religion and Health*.

[51] Asgari M., Pouralizadeh M., Javadi Pashaki N. (2022). Perceived spiritual care competence and the related factors in nursing students during Covid-19 pandemic. *International Journal of Africa Nursing Sciences*.

[52] Zhang X., Ba L., Xu J. (2023). Analysis of the current status of community nurses’ spiritual care competencies and the factors: a descriptive cross-sectional analysis. *Nursing Open*.

[53] Jing W., Jing Z., Yali L., Xiaomin X., Hongjuan S. (2022). A study on the mediating effect of psychological capital on learning motivation and perceived professional benefits in nursing interns. *Journal of Nursing Administration*.

[54] Yi Q. F., Yan J., Zhang C. J., Yang G. L., Huang H., Yang Y. (2022). The experience of anxiety among Chinese undergraduate nursing students in the later period of their internships: findings from a qualitative study. *BMC Nursing*.

[55] Taylor R., Thomas-Gregory A., Hofmeyer A. (2020). Teaching empathy and resilience to undergraduate nursing students: a call to action in the context of Covid-19. *Nurse Education Today*.

[56] Swift A., Banks L., Baleswaran A. (2020). COVID-19 and student nurses: a view from England. *Journal of Clinical Nursing*.

[57] Cuicui Y., Rumei L., Ni Y., Baodi Q. (2023). Career success and its determinants among hospice nurses. *Journal of Nursing Science*.

[58] Jinxia J., Haiyan S., Xiaoping Z., Meimei T. (2020). Perspectives of professional benefits among emergency nurses: aqualitative study. *Journal of Nursing Science*.

[59] Chen H. M., Liu C. C., Yang S. Y., Wang Y. R., Hsieh P. L. (2021). Factors related to care competence, workplace stress, and intention to stay among novice nurses during the coronavirus disease (COVID-19) pandemic. *International Journal of Environmental Research and Public Health*.

[60] Chang Y. C., Yeh T. F., Lai I. J., Yang C. C. (2021). Job competency and intention to stay among nursing assistants: the mediating effects of intrinsic and extrinsic job satisfaction. *International Journal of Environmental Research and Public Health*.

[61] Rodríguez-García M. C., Márquez-Hernández V. V., Granados-Gámez G., Aguilera-Manrique G., Gutiérrez-Puertas L. (2021). Magnet hospital attributes in nursing work environment and its relationship to nursing students’ clinical learning environment and satisfaction. *Journal of Advanced Nursing*.

[62] Guo Y. F., Cross W. M., Lam L., Plummer V., Wang X. X., Wang S. S. (2021). Association between psychological capital and spiritual care competencies of clinical nurses: a multicentre cross-sectional study. *Journal of Nursing Management*.

[63] Chiang Y. C., Lee H. C., Chu T. L., Han C. Y., Hsiao Y. C. (2020). A spiritual education course to enhance nursing students’ spiritual competencies. *Nurse Education in Practice*.

[64] Jones K. F., Paal P., Symons X., Best M. C. (2021). The content, teaching methods and effectiveness of spiritual care training for healthcare professionals: a mixed-methods systematic review. *Journal of Pain and Symptom Management*.

[65] van der Riet P., Levett-Jones T., Courtney-Pratt H. (2018). Nursing students’ perceptions of a collaborative clinical placement model: a qualitative descriptive study. *Nurse Education in Practice*.

[66] Juan G., Xiaohua S., Zihang C., Hongtao C., Yaoyue L. (2020). Current situation of nursing interns’ perception of stress and lts lmpact on professional ldentity during COVID-19. *Journal of Hunan University of Chinese Medicine*.

[67] Liu S., Duan X., Han P., Shao H., Jiang J., Zeng L. (2022). Occupational benefit perception of acute and critical care nurses: a qualitative meta-synthesis. *Frontiers in Public Health*.

